# Resection arthrodesis for the management of aggressive giant cell tumor of the distal femur

**DOI:** 10.4103/0019-5413.44432

**Published:** 2009

**Authors:** Ayman Abdelaziz Bassiony, Mohamed Abdelrahman, Amr Abdelhady, Mohamed Kamal Assal

**Affiliations:** Orthopaedic Department, Ain Shams University, Demerdash Hospital, Abbasseia Square, Cairo, Egypt

**Keywords:** Giant cell tumor, intra medullary nail, resection arthrodesis

## Abstract

**Background:**

Giant cell tumors (GCTs) of bone are aggressive benign tumors. Wide resection is reserved for a small subset of patients with biologically more aggressive, recurrent, and extensive tumors. Wide resection and mobile joint reconstruction are preferable for treating tumors around the knee. In certain situations, resection arthrodesis or an amputation is suggested. In this prospective study we report the outcome of 8 patients of aggressive GCT of lower end of femur treated with resection arthrodesis.

**Materials and Methods:**

Eight patients with mean age of 37.25 years (range 30–45 years) with Campanacci Grade III (Enneking stage III) giant cell tumors at the distal femur were treated with wide resection and arthrodesis using dual free fibular graft and locked intramedullary nail from January 2003 to January 2008. There were four males and four females patients. The mean follow-up was 48.75 months (range 30–60 months). The functional evaluation was done using the standard system of musculoskeletal tumor society with its modification developed by Enneking *et al.*

**Results:**

At the final follow up the functional score ranged from 20 to 27 out of total score of 30. Graft union was achieved in all cases in a duration mean of 14.5 months (range 12-20 months).One case required secondary bone graft due to delayed union, and one case had superficial wound infection which healed on systemic antibiotics. At final followup, all the patients were disease free.

**Conclusion:**

Wide resection and arthrodesis in aggressive GCTs of the distal femur with involvement of all muscle compartments is a good treatment option. Resection arthrodesis offers a biological reconstruction alternative to amputation in a special group of patients when extensive resection precludes mobile joint reconstruction.

## INTRODUCTION

Giant cell tumor (GCT) is a benign aggressive tumor of bone.^1^ It most frequently occurs in the distal end of the femur and the proximal end of the tibia.^1^ Most of the patients are between 20 and 45 years of age.^2^ The World Health Organization has classified GCT as an aggressive, potentially malignant lesion.Its histogenesis is uncertain.^3^,^4^

The available surgical options range from curettage to wide resection with suitable reconstruction.^5^ Although extended curettage has produced good results in well-contained GCTs, it has not done so in tumors with cortical breach and large soft tissue masses. Hence, wide resection is reserved for a small subset of patients with biologically more aggressive, recurrent, and extensive tumors.^6^

The ideal reconstruction of the defect created after en block resection of the tumor is still a subject of debate. Endoprosthetic replacement incurs a high cost, requires adequate motor reconstruction and repeated surgeries. Massive allografts are widely used in many centers. However, it requires substantial time and money, and for a variety of reasons, it is not available in many countries.^7^ An arthrodesis is less attractive initially, but once it is achieved, it provides a stable limb, and the patient is unlikely to require revision surgery. Resection arthrodesis can be done by several methods including intercalary autograft using dual fibular graft to bridge the intercalary defect after en bloc resection and callus distraction with Ilizarov method.^8^

In this prospective case series, we report the outcome in eight patients with aggressive GCT of the lower end of femur, treated with wide resection and arthrodesis of the knee, and report the radiological and functional outcome at a mean follow-up of 4 years.

## MATERIALS AND METHODS

This prospective study included eight patients with aggressive giant cell tumor of the distal femur, who were operated between January 2003 and May 2005. All participating surgeons used the same technique of wide resection and reconstruction. There were four male and four female patients. Ages ranged from 30 to 45 years (mean of 37.25 years). The mean follow-up was 48.75 months (range, 30–60 months) [Table 1]. One case (case 1) presented to us, after she had been treated at other institution with curettage and cementation, with extensive local recurrence and pathological fracture. Another patient (Case 2) presented to us after having undergone intramedullary nailing for undiagnosed pathological fracture distal femur.

**Table 1:** Details of the study group

Case	Age	Sex*	Follow-up (M)	Graft union (M)	Functional score	Complications
1	38	F	60	12	90	None
2	30	M	40	15	90	None
3	38	M	30	20	80	Non union
4	30	F	60	13	76	Wound infection
5	36	M	46	12	83	None
6	36	F	54	12	66	None
7	45	F	48	18	76	None
8	45	M	52	12	66	None

* F = Female, M = Male

All patients underwent staging studies that included plain radiography, computed tomography (CT), magnetic resonance imaging (MRI), and chest CT. The aim of these studies was to determine the extent of the soft tissue component, its relation with the neurovascular bundle, breach of the articular surface, the intramedullary extent of the disease, and planning a precise resection length.

Campanacci's staging system for giant cell tumor of bone^9^ was used for cortical breach. According to this system, all cases were graded as stage III. Enneking^10^ staging was used preoperatively. According to this system, all cases were found to be in Enneking stage III.

If the clinical presentation and the imaging studies were compatible with diagnosis of a classic benign giant cell tumor of bone, the biopsy (frozen section) and surgery were performed during the same session (six cases). In case of atypical clinical or radiologic presentation, either CT-guided core needle (one case) or open incisional biopsy was performed, and surgery was delayed until histopathologic evaluation had been completed (one case).

### Operative procedure

A long medial incision that begins in the mid-thigh, crossing the knee joint along the medial parapatellar area and distal to the tibial tubercle, extending gently posterior to the inferior border of the pes muscles was made. The biopsy site was included, with a 1-cm margin in all directions. Fasciocutaneous flaps were developed. The popliteal space was approached by detaching or retracting the medial hamstrings. The superficial femoral artery was identified within the sartorial canal. The length of the resection was measured from the medial joint line to the correct area on the femur and then marked. All the remaining soft tissue at the level of transection was cleared. The tumor was resected with all involved muscles. Extraarticular resection was done in all cases followed by knee preparation for fusion. The resulting defect ranged from 14 to 17 cm with a mean of 15.87 [Table 2]. Reconstruction was done by dual fibular autografts spanning the defect. The length of the graft ranged from 16 to 18 cm with a mean of 16.8 cm [Table 2]. Fixation was done by locked intramedullary knee fusion nail [Huckstep nail (Downs Surgical, Sheffield, United Kingdom)]. The nail was applied through retrograde insertion. The nail is inserted 5–6 cm beyond the isthmus of the femur and tibia after tumor resection, and each segment was fixed with locking screws. We used only the short retrograde nail with the length of 46 cm, [Table 2].

**Table 2:** The length of the defect, the graft, and the nail

Case	The gap after resection (cm)	The length of the graft (cm)	The length of the nail (cm)
1	17	18	46
2	15	16	46
3	16	17	46
4	17	18	46
5	17	18	46
6	16	17	46
7	15	15	46
8	14	15	46

**Figure 1:**
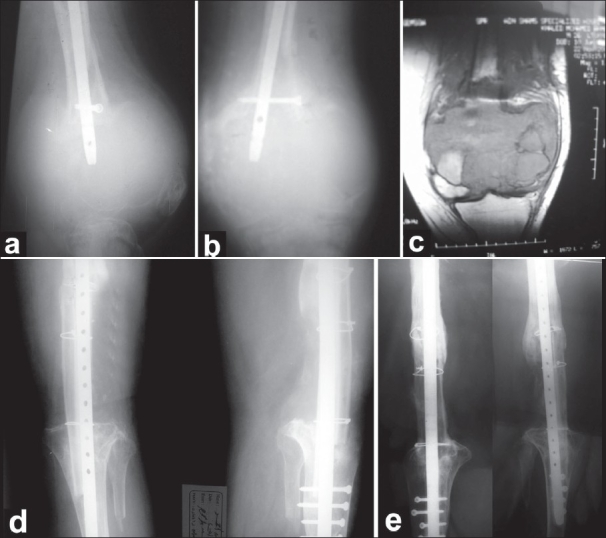
Case 2. (a and b) Preoperative X-rays showing extensive bone destruction at the distal femur with the intramedullary nail inside. (c) MRI showing extensive soft tissue involvement of all compartments. (d) Postoperative X-rays. (e) Follow-up X-rays after 48 months showing graft union and hypertrophy.

In case 2, the patient presented with intramedullary nail used for fixation of undiagnosed pathological fracture. The nail had to be removed through a separate proximal lateral incision, and the entire medulla was irrigated with phenol [Figure 1].

Postoperatively, the patients were examined every week for the first month for the detection of early complications as infection and then monthly for the first year for early detection of any local or systemic recurrence and progression of graft incorporation. We used the system of Hsu *et al* (1997) for assessment of graft union both proximally and distally which require unequivocal radiographic evidence of bone healing of graft and osteotomy sites including uninterrupted external bony borders between the fibular graft and the recipient bone, obscured or absent osteotomy lines at both junctions, and clinical resumption of normal function and weight –bearing without discomfort.^10^ At every visit, the patients were examined clinically and radiologically. Then, the patients were examined every 6 months for the rest of the follow-up period. The patients were allowed partial weight-bearing with a long leg cast at the earlier sign of union. The patients however returned to vigorous activity only after full union of the graft.

## RESULTS

At the end of the follow-up, all patients were alive and were free from any signs of local or systemic recurrence. The functional evaluation was performed using a modified system of the Musculoskeletal Tumor Society.^11^ At the final follow-up, the functional score ranged from 20 to 27 out of a total 30 i.e. a mean score of 78.3% (range 66-90%).

**Figure 2:**
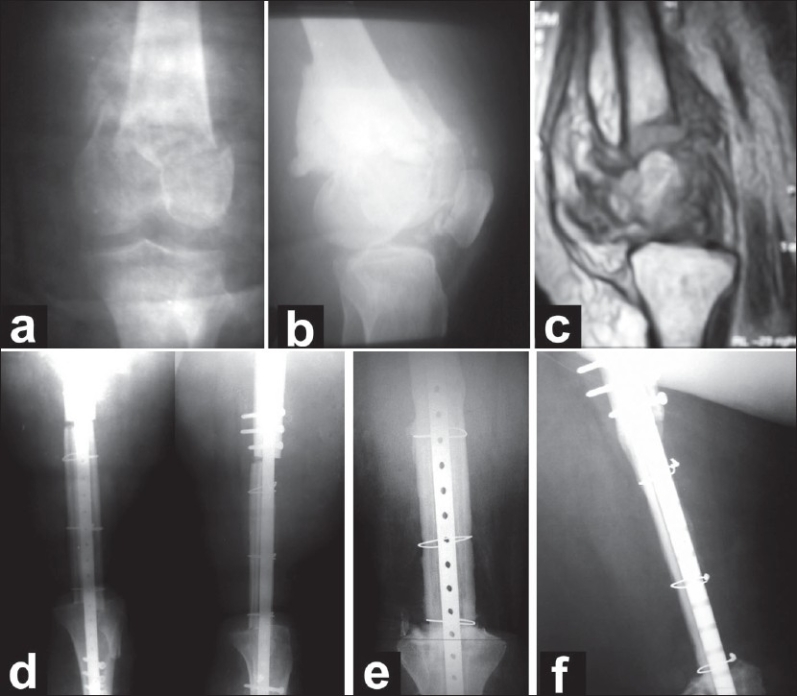
Case 1. (a and b) Preoperative X-rays showing osteolytic lesion of the distal femur with pathological fracture and bone cement. (c) MRI showing extensive soft tissue involvement and pathological fracture. (d) Immediate postoperative X-rays. (e and f) X-rays after 12 months showing full union of the graft at both the proximal and distal end.

Radiological union of the reimplanted segment proximally and distally was assessed according to the method proposed by Hsu *et al.*^10^ as discussed earlier. According to this system, the graft united proximally and distally [Figure 2] in all patients with a mean of 14.5 months (range 12–20 months). In one patient, additional grafting at the proximal host graft junction site with cancellous bone graft from the iliac crest was required at 16 months postoperative, and 4 month later, full union was achieved. Superficial wound infection occurred in one patient (case 4) and was treated by systemic antibiotics. No complications of graft resorption, implant failure, or neurovascular complications were encountered.

## DISCUSSION

Giant cell tumor is an infrequent and unpredictable lesion.^2^,^12^,^13^ Other authors have generally failed to ascertain the biological or histological parameters that either determine the prognosis or indicate the best treatment.^14^–^15^

The principal treatment modality of this locally aggressive benign tumor is surgery.^6^ The adequate removal of the tumor does minimize the risk of recurrence.^16^,^17^ The surgical treatment of this tumor has always been controversial, with the desired treatment being a balance between adequate removal and retention of function.^18^ The available surgical options include curettage with bone grafting, extended curettage using chemical cauterization/cryosurgery with bone grafting, cementation with or without bone grafting, and wide resection with suitable reconstruction. Although extended curettage has produced good results in well-contained GCTs, it has not done so in tumors with cortical breach and large soft tissue masses. Hence, wide resection becomes indicated.^6^

The defects resulting from tumor resection can be reconstructed using various approaches.^19^–^20^ At present, there is no single generally accepted satisfactory method for reconstructing massive osseous and soft tissue defects after wide resection of a malignant or aggressive bone tumor. When patients or orthopedists are given a choice, they prefer limb salvage procedures that allow for knee motion.^21^–^22^ However, functional mobile knee reconstruction requires active knee extension. When the quadriceps is resected with the tumor, the patient should have arthrodesis.^23^

Several methods of resection arthrodesis were described. Yadav advocates the use dual fibular graft to bridge the intercalary defect after en bloc resection.^24^ He reported 52 patients (GCT 37 and osteosarcoma 15) where the size of gap ranged from 9 to 24 cm. In the later part of his series, he advocates the use of Kirschner wires inside the long grafts to help maintaining the continuity of the graft when a stress fracture occurs.

Kapukaya *et al* described limb reconstruction with the callus distraction method in seven cases of tumors of the distal femur. The defect after tumor resection ranged from 8 to 20 cm.^25^ Tsuchiya *et al* described the use of the Ilizarov technique for management of subarticular defects after en bloc resection or curettage and phenol cauterization in GCT of the proximal tibia in five patients. The mean length of bone defect was 5.7 cm, and the mean duration of external fixation was 233 days. The advantages of this method include the lack of graft rejection, the reattachment of ligaments and tendon to the bone, the prevention of articular collapse, early movement of the knee, and ankle joint and early weight-bearing. Disadvantages include the long duration of external fixator application, pin tract infection, wire breakage, and frustration of patients due to the long duration of treatment.^26^

In our prospective clinical study, eight patients with Campanacci grade III giant cell tumor of the distal femur were treated using resection arthrodesis by dual nonvascularized fibular graft and knee fusion nail. At a mean follow-up of 4 years, all the patients were alive and free from any signs of local or systemic recurrence, with the graft united proximally and distally in all patients. Similar rates of union (100%) following resection arthrodesis of the knee in various tumors have been reported by other workers.^24^–^26^ They used a hemicortical graft from the available bone (femur or tibia in cases with tibial and femoral tumors respectively). A good functional outcome has been reported by these workers following knee arthrodesis in these cases

Direct comparison of the results of resection arthrodesis with endoprosthetic reconstruction in our series is not appropriate due to the differences in the disease processes and indications. However, all our patients achieved good functional results, which is comparable to that of endoprosthetic patients (78.3%). This result confirms the study conducted by Harris *et al*,^27^ who reported that patients functioned similarly and walked with comparable velocities, efficiencies, and rates of consumption of oxygen whether they had been treated with arthrodesis or arthroplasty. Patients who received an arthrodesis had a more stable limb and performed the most demanding physical work, but they had difficulty in sitting. However, endoprosthetic patients have to live a more sedentary life due to a feeling of weakness and instability.

Wide resection is indicated in aggressive GCT of the distal femur with extensive soft tissue involvement. Resection of all involved muscle compartments is essential to obtain proper safety margin. This will make motor reconstruction of mobile endoprosthesis impossible, and consequently, arthrodesis will be a viable option. Arthrodesis can be achieved by the use of fibular grafts and intramedullary knee fusion nail with good to excellent results. An immovable knee in good alignment and functional position is considered to be an appropriate sacrifice to achieve a stable, pain-free limb.
